# *Wolbachia*-Driven Memory Loss in a Parasitic Wasp Increases Superparasitism to Enhance Horizontal Transmission

**DOI:** 10.1128/mbio.02362-22

**Published:** 2022-10-10

**Authors:** Jin-Cheng Zhou, Xu Zhao, Liang-Xiao Huo, Dan Shang, Hui Dong, Li-Sheng Zhang

**Affiliations:** a College of Plant Protection, Shenyang Agricultural University, Shengyang, Liaoning, People’s Republic of China; b Institute of Plant Protection, Chinese Academy of Agricultural Sciences, Beijing, People’s Republic of China; University of Chicago

**Keywords:** Trichogramma, *Wolbachia*, behavioral manipulation, memory loss, superparasitism

## Abstract

Horizontal transmission of the endosymbiont, *Wolbachia*, may occur during superparasitism when parasitoid females deposit a second clutch of eggs on a host. *Wolbachia* may increase the superparasitism tendency of Trichogramma wasps by depriving their memory. To test this hypothesis, we investigated the effects of conditioning experience and memory inhibitors (actinomycin D [ACD] and anisomycin [ANI]) on memory capacity, and expressions of memory-related genes (*CREB1* and *PKA*), and superparasitism frequency of *Wolbachia*-infected (TDW) and uninfected (TD) lines of Trichogramma dendrolimi after conditioning with lemon or peppermint odor. We detected the presence of *Wolbachia* in eggs, larvae, pre-pupae, pupae, and adults of Trichogramma by using fluorescence *in situ* hybridization. The results showed that TDW females had a more reduced memory capacity than TD females after conditioning. Compared with TD females, TDW females showed a higher proportion of superparasitism and a downregulation of *CREB1* and *PKA* genes after conditioning. TD females fed ACD or ANI showed a higher tendency for superparasitism and a downregulation of *CREB1* and *PKA*, along with memory loss after conditioning than TD females fed honey solution only. The presence of *Wolbachia* was detected in the anterior region of the larva, pre-pupa, and pupa, but was not found in the head of the adult. The results provide evidence of host behavioral manipulation of *Wolbachia* by depriving memory of host Trichogramma wasps based on Poulin’ s criteria. These host behavioral changes led by *Wolbachia* may be caused by the virulence of *Wolbachia* on the nervous system of the host.

## INTRODUCTION

The endosymbiotic bacteria, *Wolbachia*, live widely within cells of insects and other arthropods. *Wolbachia* are predominantly transmitted vertically from host mother to offspring through eggs and often manipulate the reproduction of host arthropods in diverse ways, such as by inducing parthenogenesis, cytoplasmic incompatibility (CI), male killing (MK), and feminization (1). However, *Wolbachia* can also be horizontally transmitted among host individuals or even across species boundaries (1, 2). In nature, horizontal transfer (HT) of *Wolbachia* may occur when host individuals are in close contact, as *Wolbachia* can briefly persist outside host cells before traversing the cell membranes of the host (3, 4). Such close contact among parasitoid offspring individuals is expected to occur during superparasitism, i.e., when parasitoid females deposit a clutch of eggs on a host previously parasitized by the same parasitoid species (5, 6). In this situation, *Wolbachia*-infected parasitoid offspring and uninfected parasitoid offspring may share the same host (5, 7). The first natural case of high rates of HT in Trichogramma wasps was found in the egg parasitoid, Trichogramma kayki, when *Wolbachia*-infected and uninfected parasitoid eggs were deposited into the same host egg (8). Some of the uninfected T. kaykai larvae may acquire the infection of *Wolbachia* under such condition. Huigens et al. (9) further tested the intraspecific HT of *Wolbachia* in several Trichogramma species under superparasitism conditions, and detected intraspecific HT in Trichogramma kaykai and Trichogramma deion, but did not detect HT in Trichogramma atopovirilia. Our previous study also observed the intraspecific HT of *Wolbachia* in Trichogramma dendrolimi when the infected offspring egg was deposited into a host parasitized by an uninfected wasp for 1, 8, or 16 h; however, the HT was not detected when the infected offspring egg was deposited at 32 h after the host was parasitized by the uninfected wasp (10). This implies that *Wolbachia* may gain the opportunity for horizontal transmission among Trichogramma offspring by causing superparasitism.

Superparasitism is often viewed as a maladaptive mistake of wasps, as the parasitoid wasp is expected to allocate an optimal clutch size in a host to maximize the fitness according to Lack’s hypothesis (11–13). Interestingly, some symbionts can increase the superparasitism tendency of their host (parasitoid wasps), by allowing them to transmit to parasitoid offspring that share the same host insect (9, 14, 15). Thus, a question is immediately proposed that whether superparasitism behavior is a behavior modification induced by the symbiont to favor symbiont transmission, and viewed as “infectious behavior” (14–16). For example, a male-killing heritable symbiont, Arsenophonus nasoniae, benefits from superparasitism spreading in the offspring of the pupal parasitoid, Nasonia vitripennis (15). In Leptopilina boulardi, parasitoid females infected with the L. boulardi Filamentous Virus (LbFV) showed a higher tendency for superparasitism than their uninfected counterparts (17, 18). Farahani et al. (19) reported that *Wolbachia*-infected T. brassicae wasps superparasitized their hosts more often than their uninfected counterparts. Similarly, our previous studies also showed that T. dendrolimi females infected by *Wolbachia* exhibited a higher tendency of superparasitism than the uninfected females (10, 20). Although *Wolbachia*-infected females of T. brassicae (19) and T. dendrolimi (10, 20) exhibited a higher tendency for superparasitism than uninfected females, the mechanism behind this phenomenon is still unknown.

Poulin (21) proposed a series of criteria for testing whether the behavioral changes of a host are due to the adaptations of a parasite that is trying to enhance its transmission (“host manipulation hypothesis”): (i) the behavioral changes must be complex (because complex traits are unlikely to arise by chance); (ii) the behavioral changes must show purposeful design for a particular function; (iii) the behavioral changes are more likely to be an adaption if these changes arise in several lineages or related species (high superparasitism tendency of *Wolbachia-*infected Trichogramma females has been found in Trichogramma brassicae and T. dendrolimi); (iv) the manipulation should increase the transmission of the parasite. The higher superparasitism tendency in *Wolbachia-*infected Trichogramma females appears to fulfill the third criteria of the host manipulation hypothesis (10, 19, 20). Clearly, the most important criteria is that behavioral manipulation should be aimed at increasing the fitness of the parasite (criteria 4). The occurrence of superparasitism would result from benefit conflicts between *Wolbachia* and host wasps. For parasitoid wasps, the fitness of parasitoid larvae is entirely dependent on the quality of the host for their development (22). Superparasitism often results in the intraspecific competition of parasitoid larvae in most cases, leading to offspring fitness costs or even the killing of offspring (5). This may come at the cost of a reduction in the vertical transmission of the parasite (23). Broadly speaking, the outcome of intrinsic intraspecific competition depends on the host usage strategy of parasitoid larvae. In “solitary” parasitoids (i.e., only one parasitoid offspring can successfully develop on or inside a host), the larva is seeking to monopolize the host resources; if more than one eggs have been laid in the same host, no more than one parasitoid offspring is expected to survive from the ensuing larval competition (5, 23). Trichogramma wasps can be viewed as a gregarious species, where more than one parasitoid offspring can successfully emerge from a host. The offspring of gregarious species can often survive under superparasitism conditions, although the offspring wasps may exhibit a small body size or short longevity (5, 22). Thus, *Wolbachia* may gain an opportunity for HT among these survival offspring.

Generally, the high tendency of superparasitism can be attributed to 2 broad explanations. One is that superparasitism is an adaptive strategy for short-lived wasps that rarely encounter healthy hosts in field conditions (22, 24, 25). This explanation predicts that wasps are less willing to accept a parasitized host when healthy hosts are common in the environment. Superparasitism is found more frequently when a wasp carries large supplies of unlaid eggs and is certain to die in a limited time (22, 25). However, the high tendency of superparasitism of *Wolbachia*-infected Trichogramma has been observed under laboratory conditions, where a certain number of hosts were supplied to newly emerged infected or uninfected wasps (10, 19, 20). This implies that the high tendency of superparasitism in *Wolbachia*-infected Trichogramma may be attributed to other factors. An alternative interpretation is that frequent superparasitism by *Wolbachia*-infected Trichogramma may be a result of the breakdown in the mechanism of host discrimination (25). Generally, parasitoid females prevent superparasitism by labeling their host with host marking pheromones (HMP) (13, 22, 25). Newly enclosed parasitoid females experience both healthy (unparasitized) and parasitized hosts, enabling them to discriminate between the 2 (24, 25). Previous studies reported that *Wolbachia*-infected Trichogramma exhibited a shorter memory than natural uninfected Trichogramma (26, 27). Thus, a hypothesis is proposed that, based on observations of the high tendency of superparasitism and memory loss in *Wolbachia*-infected Trichogramma, *Wolbachia* infection may select for reducing memory of Trichogramma females, causing a breakdown in the mechanism of host discrimination, and resulting in an increased tendency for superparasitism. This is called the “memory retention hypothesis” (27, 28).

To test the hypothesis, *Wolbachia*-infected (TDW) and uninfected (TD) lines of T. dendrolimi were used as the model. This study followed the definition of superparasitism by van Dijken and Waage (29). Superparasitism is defined as the deposition of a clutch of eggs in or on a host that has already been parasitized by a female of the same species (29). Trichogramma wasps are often viewed as a facultative gregarious parasitoid species and have been widely used as an effective biological control agent against many lepidopteran pests in agriculture and forestry (30, 31). T. dendrolimi females often allocate tens of eggs on a larger lepidopteran egg (e.g., Antheraea pernyi and Samia cynthia) (31–33), and often allocate a single egg on a smaller lepidopteran egg (e.g., Corcyra cephalonica) (34). In this study, C. cephalonica eggs were used as the host eggs for a more exact determination of superparasitism (28).

Here, we examined the effects of *Wolbachia* infection on the memory of *Wolbachia*-infected (TDW) and uninfected (TD) females of T. dendrolimi after conditioning with lemon odor or peppermint odor and an egg card as a reward using a modified version of the methodology described by Farahani et al. (26, 27). The memory inhibitors, actinomycin D (ACD) (35) and anisomycin (ANI) (36), were applied to block the memory of TD and TDW females. The memory capacity of Trichogramma females was estimated by the proportion of choice (PCO) and the proportion of residence time (PRO) for the conditioning odor with a 24 h time interval after learning. The choice of the wasp was determined when the wasp crossed a line located 30 mm away from a tunnel pumping conditioning or control odor. The residence time of the wasp was defined as the amount of time spent in the area with conditioning or the control odor before the wasp made the choice. Thereafter, we tested the memory capacity, frequency of superparasitism, and the expression of the indicator genes (*CREB1* and *PKA*) related to memory formation (37, 38) in TD and TDW females with memory inhibition and *Wolbachia* infection. The results of this study are expected to bridge the gaps in understanding the behavioral mechanisms of *Wolbachia*-induced superparasitism in Trichogramma wasps and to highlight the manipulative effects of symbionts on host parasitoids.

## RESULTS

To avoid side effects of ANI and ACD on the survival of Trichogramma females, a 15% honey solution mixed with 0.008 mg/mL ACD or 0.1 mg/mL ANI was used to block the memory of Trichogramma females according to the results of pre-experiments (Text S1 and Fig. S1). Compared with the experienced female wasps in TDW line, the experienced female wasps in TD line exhibited a higher memory capacity (a higher tendency to the conditioning odor) at 24 h after conditioning, but this was not true for those wasps at 12 and 36 h after conditioning (Text S1, and Fig. S2 and S3).

10.1128/mbio.02362-22.3 FIG S1 Cumulative mortality risk of *Wolbachia*-infected (TDW) and uninfected (TD) *Trichogramma* influenced by different concentrations of ACD and ANI. This experiment was performed once. Download FIG S1, TIF file, 2.2 MB. Copyright © 2022 Zhou et al. 2022 Zhou et al. https://creativecommons.org/licenses/by/4.0/ This content is distributed under the terms of the Creative Commons Attribution 4.0 International license.

10.1128/mbio.02362-22.4 FIG S2 Proportion of choice for conditioning odor, lemon (A) or peppermint (B), and proportion of choice for control odor in naive females and experienced females of *Wolbachia*-infected (TDW) and uninfected (TD) *Trichogramma* at 12, 24, and 36 h after conditioning. Error bars indicate 95% confidence interval. The different uppercase letters indicate significant differences among TD females subjected to different treatment. The different lowercase letters indicate significant differences among TDW females subjected to different treatments. “ns” and “*” indicate nonsignificant differences and significant differences at *P < *0.05, respectively, between TD and TDW females. This experiment was performed once. Download FIG S2, TIF file, 2.1 MB. Copyright © 2022 Zhou et al. 2022 Zhou et al. https://creativecommons.org/licenses/by/4.0/ This content is distributed under the terms of the Creative Commons Attribution 4.0 International license.

10.1128/mbio.02362-22.5 FIG S3 Proportion of residence time for conditioning odor, lemon (A) or peppermint (B), and proportion of choice for control odor in naive females and experienced females of *Wolbachia*-infected (TDW) and uninfected (TD) *Trichogramma* at 12, 24, and 36 h after conditioning. Error bars indicate 95% confidence interval. The different uppercase letters indicate significant differences among TD females with different treatments. The different lowercase letters indicate significant differences among TDW females with different treatments. “ns” and “*” indicate nonsignificant differences or significant differences at *P < *0.05, respectively, between TD and TDW females. This experiment was performed once. Download FIG S3, TIF file, 2.4 MB. Copyright © 2022 Zhou et al. 2022 Zhou et al. https://creativecommons.org/licenses/by/4.0/ This content is distributed under the terms of the Creative Commons Attribution 4.0 International license.

### Effects of *Wolbachia* infection and memory blocking on memory of Trichogramma females.

**FIG 1:**
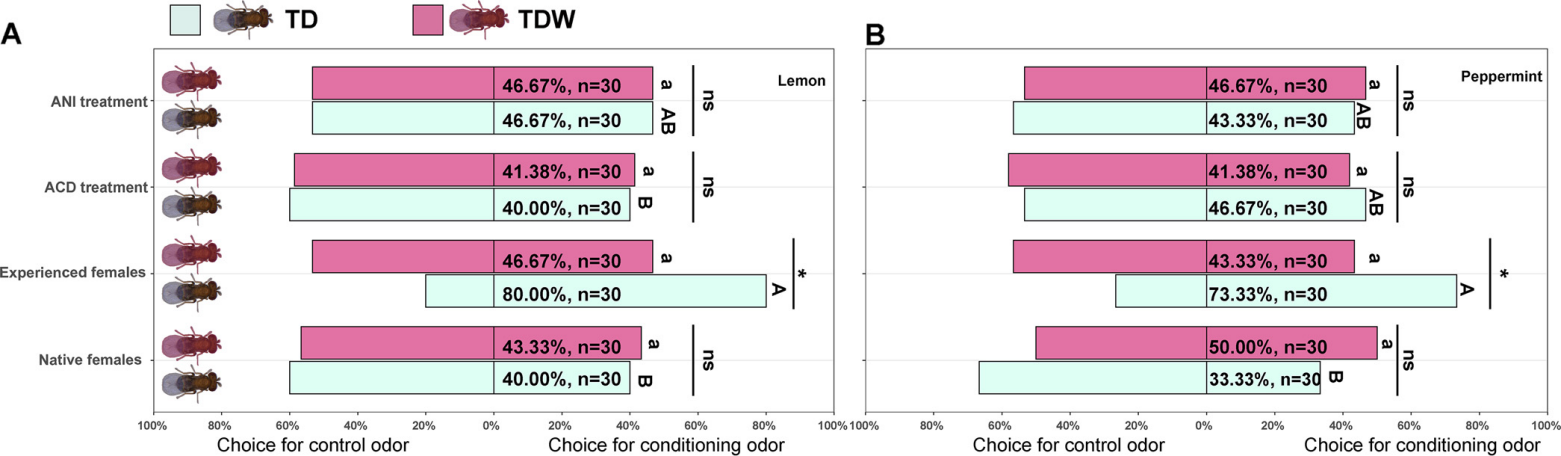
Proportion of choice for conditioning odor, lemon (A) or peppermint (B), and proportion of choice for control odor in naive females and experienced females of TD and TDW line fed honey solution only, ACD or ANI at 24 h after conditioning. Error bars indicate 95% confidence intervals. The different uppercase letters indicate significant differences among TD females subjected to different treatments. The different lowercase letters indicate significant differences among TDW females subjected to different treatments. “ns” and “*” indicate nonsignificant differences and significant differences at *P < *0.05, respectively, between TD and TDW females. This experiment was performed once.

**FIG 2:**
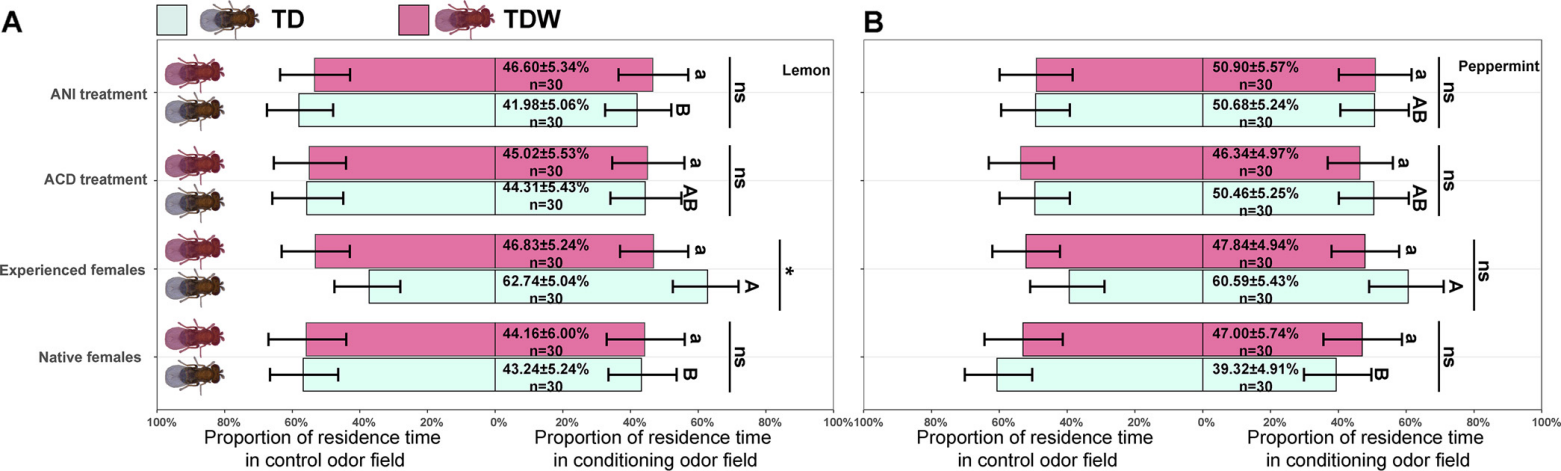
Proportion of residence time for conditioning odor, lemon (A) or peppermint (B), and proportion of choice for control odor in naive females and experienced females of TD and TDW line fed honey solution only, ACD or ANI at 24 h after conditioning. Error bars indicate 95% confidence interval. The different uppercase letters indicate significant differences among TD females subjected to different treatments. The different lowercase letters indicate significant differences among TDW females subjected to different treatments. “ns” and “*” indicate nonsignificant differences or significant differences at *P < *0.05, respectively, between TD and TDW females. This experiment was performed once.

The PCOs of experienced TD females were significantly higher (*P = *0.0014 [Lemon]; *P = *0.016 [Peppermint]) than the theoretical value of 50%, but these were not true in case of experienced TDW females (*P = *0.86 [Lemon]; *P = *0.58 [Peppermint]). Regardless of *Trichogramma* line, the PCOs of naive females, experienced females fed ACD, and experienced females fed ANI were not different to the theoretical value of 50%. The PCOs (*z *= 2.55, *P = *0.011 [Lemon]; *z *= 2.27, *P = *0.023 [Peppermint]) and PROs of experienced TD females were significantly or marginally insignificantly higher (*z *= 2.16, *P = *0.031 [Lemon]; *z *= 1.64, *P = *0.10 [Peppermint]) than those of TDW females, but these differences were not true in case of naive females, experienced females fed ACD, and experienced females fed ANI (Fig. 1 and 2). The PCOs of experienced TD females conditioned to lemon odor were significantly or marginal insignificantly higher than those of naive females (*z *= 2.99, *P = *0.015), experienced females fed ACD (*z *= 2.99, *P = *0.015), and experienced females fed ANI (*z *= 2.55, *P = *0.052). The PCOs of experienced TD females conditioned with peppermint odor were significantly higher than those of naive females (*z *= 2.96, *P = *0.016), but were insignificantly higher than those of experienced females fed ACD (*z *= 2.04, *P = *0.17), and experienced females fed ANI (*z *= 2.27, *P = *0.10). In addition, regardless of the conditioning, the PCOs of experienced TDW females were not different from those of naive females (*z *= 0.26, *P = *0.99 [Lemon]; *z *= 0.51, *P = *0.96 [Peppermint]), experienced females fed ACD (*z *= 0.40, *P = *0.98 [Lemon]; *z *= 0.11, *P = *0.9995 [Peppermint]), and experienced females fed ANI (*z *= 0, *P = *1.00 [Lemon]; *z *= 0.26, *P = *0.99 [Peppermint]) (Fig. 1).

Regardless of Trichogramma line, the PROs of naive females, experienced females fed honey solution only, experienced females fed ACD, and experienced females fed ANI were not different to theoretical value of 50%. The PROs of experienced TD females conditioned with lemon odor were significantly or marginal insignificantly (*P = *0.070) higher than those of naive females (*z *= 2.63, *P = *0.043), experienced females fed ACD (*z *= 2.44, *P = *0.070), and experienced females fed ANI (*z *= 2.83, *P = *0.024). The PROs of experienced TD females with peppermint odor conditioning were significantly higher than those of naive females (*z *= 2.69, *P = *0.036), but were insignificantly higher than those of experienced females fed ACD (*z *= 1.29, *P = *0.57), and experienced females fed ANI (*z *= 1.27, *P = *0.58). In addition, regardless of the conditioning, the PROs of experienced TDW females were not different from those of naive females (*z *= 0.34, *P = *0.99 [Lemon]; *z *= 0.11, *P = *0.9996 [Peppermint]), experienced females fed ACD (*z *= 0.24, *P = *0.995 [Lemon]; *z *= 0.21, *P = *0.997 [Peppermint]), and experienced females fed ANI (*z *= 0.030, *P = *1.00 [Lemon]; *z *= 0.40, *P = *0.98 [Peppermint]) (Fig. 2).

### Effects of *Wolbachia* infection and memory blocking on expression of *CREB1* and *PKA*.

**FIG 3:**
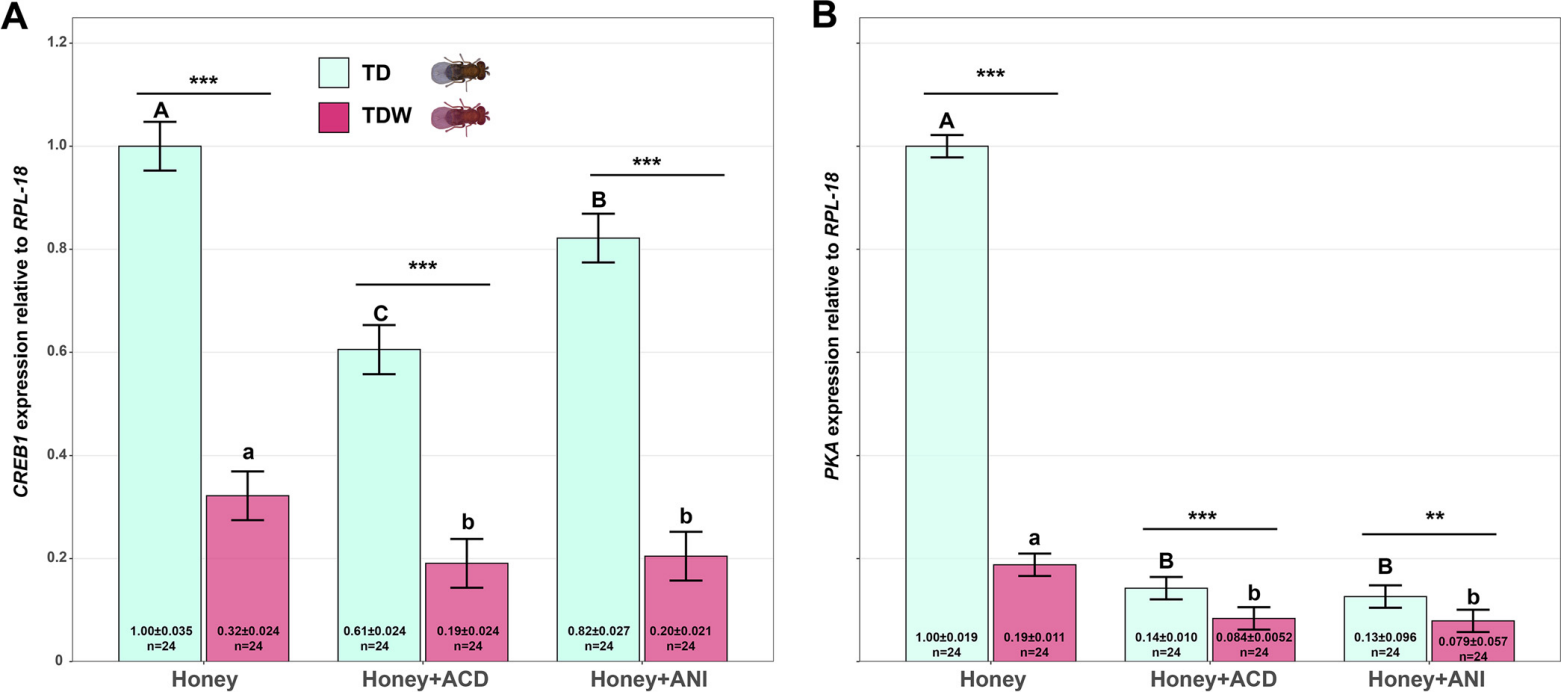
Expression level of *CREB1* (A) and *PKA* (B) of TD and TDW females fed ACD, ANI, or honey solution only (CK). Error bars indicate 95% confidence interval. The different uppercase letters indicate significant differences among TD females fed ACD, ANI, or honey solution only. The different lowercase letters indicate significant differences among TDW females fed ACD, ANI, or honey solution only. “ns,” “**,” and “***” indicate nonsignificant differences, significant differences at *P < *0.01, and *P < *0.001, respectively, between TD and TDW females. This experiment was performed thrice.

The expression levels of both memory-related genes, *CREB1* and *PKA*, were significantly affected by the interaction of the conditioning treatment and the Trichogramma line (*CREB1*: *F_2_*_,_ *_138_* = 16.29, *P < *0.001; *PKA*: *F_2_*_,_ *_138_* = 781.16, *P < *0.001). The expression levels of *CREB1* and *PKA* in TD or TDW females fed honey solution only (CK) were significantly higher than that in those fed ACD (*CREB1*: *z *= 11.55, *P < *0.001 [TD], *z *= 3.84, *P < *0.001 [TDW]; *PKA*: *z *= 54.68, *P < *0.001 [TD], *z *= 6.64, *P < *0.001 [TDW]) or ANI (*CREB1*: *z *= 5.22, *P < *0.001 [TD], *z *= 3.43, *P = *0.0017 [TDW]; *PKA*: *z *= 55.72, *P < *0.001 [TD], *z *= 6.95, *P < *0.001 [TDW]). The expression level of *CREB1* in TD females fed ACD was significantly lower (*z *= 6.33, *P < *0.001) than that in those fed ANI. Likewise, the expression level of *CREB1* in TDW females fed ACD was lower than that in those fed ANI, but the difference was insignificant (*z *= 0.41, *P = *0.91). The expression levels of *CREB1* and *PKA* in TD females fed honey solution only (*CREB1*: *z *= 19.85, *P < *0.001; *PKA*: *z *= 51.78, *P < *0.001), ACD (*CREB1*: *z *= 12.14, *P < *0.001; *PKA*: *z *= 3.74, *P < *0.001), or ANI (*CREB1*: *z *= 18.06, *P < *0.001; *PKA*: *z *= 3.01, *P = *0.0026) were significantly higher than that in TDW females with the corresponding treatment (Fig. 3).

### Effects of *Wolbachia* infection and memory blocking on superparasitism of Trichogramma females.

**FIG 4:**
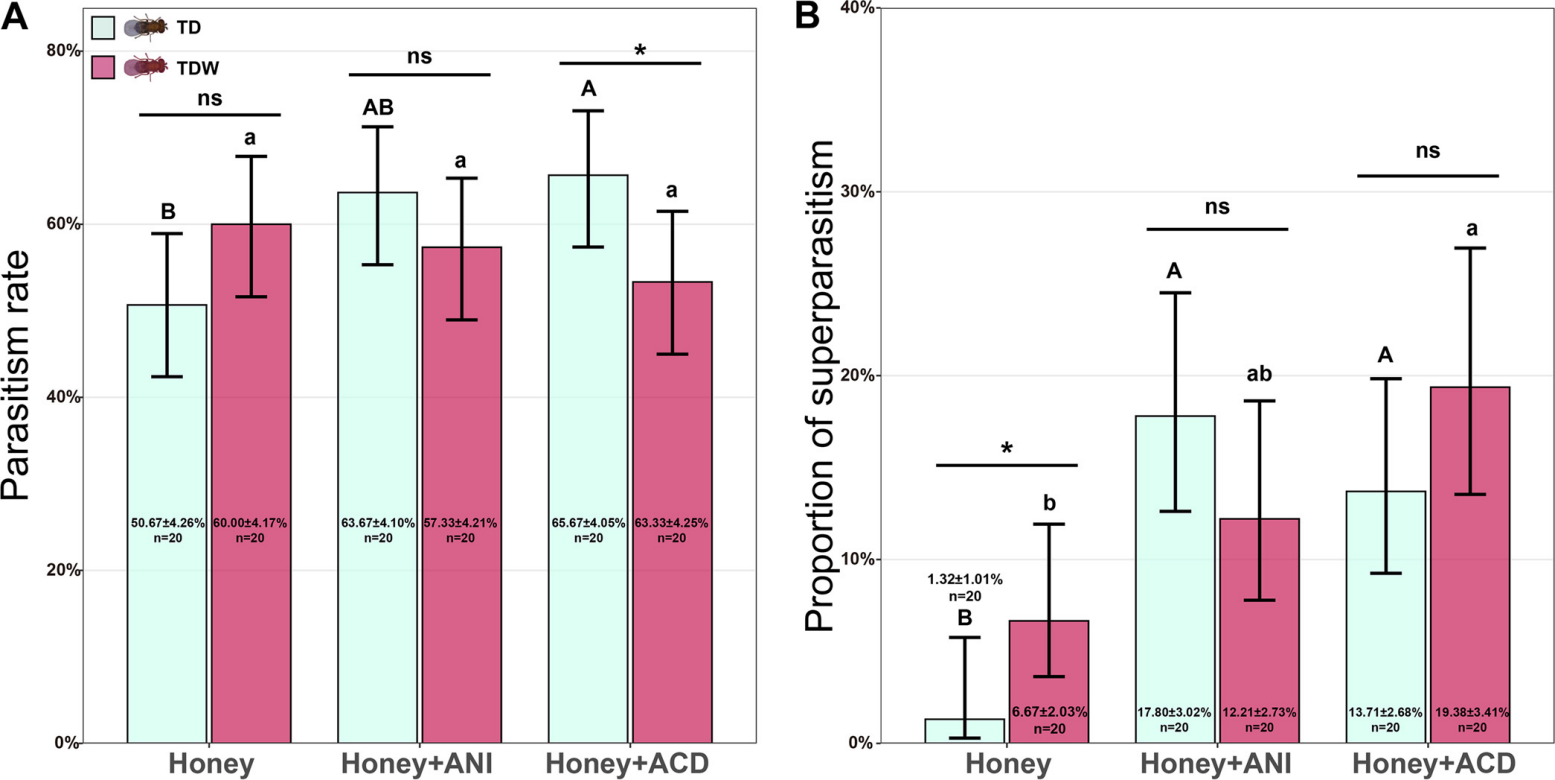
Parasitism rate (A) and proportion of superparasitism (B) of TD and TDW females fed honey solution only, ACD, or ANI at 24 h after conditioning. Error bars indicate 95% confidence interval. The different uppercase letters indicate significant differences among TD females subjected to different treatments. The different lowercase letters indicate significant differences among TDW females subjected to different treatments. “ns,” “**,” and “***” indicate nonsignificant differences, significant differences at *P < *0.01, and *P < *0.001, respectively, between TD and TDW females. This experiment was performed once.

After conditioning and a 24 h time interval, the differences in parasitism rate between TD and TDW experienced females fed honey solution only (*z *= 1.56, *P = *0.12) or ANI (*z *= 1.07, *P = *0.28) were insignificant. However, the parasitism rate by TD experienced females fed ACD was significantly higher than that by TDW experienced females (*z *= 2.08, *P = *0.038) (Fig. 4A). The proportion of superparasitism by TDW experienced females fed honey solution only (*z *= 5.97, *P < *0.001) was significantly higher than that by TD experienced females, but this difference was not true for the experienced females fed ANI (*z *= 1.35, *P = *0.18) or ACD (*z *= 1.32, *P = *0.19). Additionally, the proportion of superparasitism by TD experienced females fed ANI (*z *= 3.47, *P = *0.0015) or ACD (*z *= 3.06, *P = *0.0063) was significantly higher than that by those fed honey solution only. The proportion of superparasitism by TDW experienced females fed honey solution only was significantly lower than that by those fed ACD (*z *= 3.09, *P = *0.0057), but was not lower than that by those fed ANI (*z *= 1.61, *P = *0.24) (Fig. 4B).

### FISH detection of *Wolbachia* in the egg, larvae, pre-pupa, pupa, and adult of Trichogramma offspring.

**FIG 5:**
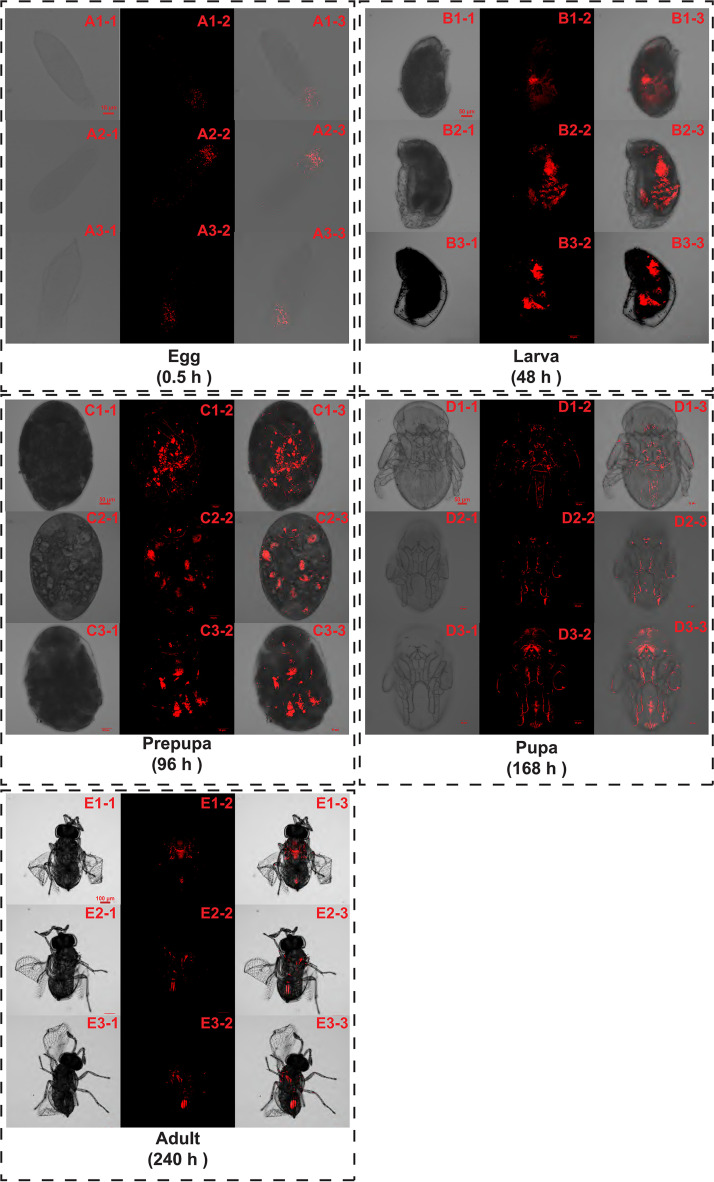
Representative images of *Wolbachia* signals in the eggs (B1) and (C1), larvae (B2) and (C2), pre-pupae (B3) and (C3), pupae (B4) and (C4), and newly emerged wasps (B5) and (C5) of host *Trichogramma*. All individuals are female. (A1) to (A5) are bright field photos. (B1) to (B5) are dark field photos stained by probes of *Wolbachia* 16S rRNA (red). (C1) to (C5) are bright field photos stained by probes of *Wolbachia* 16S rRNA (red). This experiment was performed twice.

*Wolbachia* signals were highly abundant in the posterior part of the eggs, larvae, pre-pupae, and pupae, and the abdomen of the adult. The presence of *Wolbachia* was detected in the anterior region of the larvae, pre-pupae, and pupae, but was not found in the head of the adult (Fig. 5).

## DISCUSSION

Our observations regarding the effect of *Wolbachia* infection on the memory capacity of Trichogramma were similar to the observations of Farahani et al. (27). We confirmed that *Wolbachia*-infected Trichogramma wasps showed a reduced memory capacity compared to their uninfected counterparts. Both of the indicator genes for memory formation, *CREB1* and *PKA*, were downregulated in infected females when compared to those in the uninfected counterparts. Similar to our previous studies (10, 20) and to the observation by Farahani et al. (19), we re-confirmed that infected females showed a higher superparasitism tendency than their uninfected counterparts. By blocking the memory of infected and uninfected females using ACD and ANI, we revealed the association between the higher tendency for superparasitism and the memory retention in infected females. We found that uninfected females fed ACD or ANI showed a higher tendency for superparasitism, memory loss, and downregulation of memory-related genes, which is similar to the observation in *Wolbachia*-infected females. The results support the “memory retention hypothesis”.

Though many studies have reported that some symbionts can increase the superparasitism tendency of their host (parasitoid wasps) to enhance the spread of the symbiont in the population of host wasps, the mechanisms behind this symbiont-induced behavioral change are poorly understood. According to the results of Fish detection, *Wolbachia* infection in the nervous system at the immature stages may be one underlying reason for the behavioral changes in this study. It is worth noting that *Wolbachia* signals in adult heads were weak. Similarly, our previous study also found that *Wolbachia* density was low in the heads of Trichogramma pretiosum and T. dendrolimi based on qPCR and Fish detection (39). Though *Wolbachia* signals were weak in adult heads, we still detected a significant downregulation of memory-related gene expression in infected adults compared with their uninfected counterparts. This raises an additional question: how would there be a sustained reduction in memory-related gene expression in the adult stage if *Wolbachia* is no longer present in the head? Previous studies showed that *Wolbachia* could colonize and replicate in the central nervous system (CNS) of the host insects, such as D. melanogaster (40, 41), Folsomia candida (42), Spalangia endius (43), and Eurema hecabe (44), and this may account for the effects of *Wolbachia* on host behaviors, including locomotive behaviors, sleep, feeding behaviors, mating preference and frequency, learning, and memory capacity (45, 46). The decrease in memory induced by *Wolbachia* infection is found not only in Trichogramma species, but also in Armadillidium vulgare (47). Min and Benzer (48) found that *Wolbachia* can cause a widespread degeneration of tissues, including the brain and retina. Malkeyeva et al. (49) reported that *Wolbachia-*induced a macro-autophagy in the neurons of D. melanogaster. However, without another probe for the CNS demonstrating co-localization of nervous tissue and *Wolbachia*, it is still unclear if there is a direct interaction. According to the observation on the development of a Trichogramma species, the formation of the CNS of Trichogramma telengai occurs at the late larval stage (50). The co-localization of nervous tissue and *Wolbachia* at immature stages may negatively affect the formation of the CNS. We assume that memory loss may be a side effect of the virulence of *Wolbachia* on the central nervous system. Contradicting our results, Bi et al. (51) reported that *Wolbachia* may improve the learning and memory capacity of Drosophila melanogaster and Drosophila simulans by upregulating the expression of *CREB* through microRNA. The different results may be due to the different host species and *Wolbachia* strains used in the studies. Different from Trichogramma, species such as Drosophila do not parasitize as wasps do, so memory reduction may not be beneficial. Future studies should focus on determining whether host behavioral changes are directly caused by the virulence of *Wolbachia* on the host nervous system.

Although this study reveals that *Wolbachia* may benefit from HT by inducing superparasitism through memory loss in Trichogramma wasps, the experiments were conducted under laboratory conditions. Many questions need to be answered regarding this host-parasite interaction under field conditions. For example, does superparastism negatively affects the vertical transmission of *Wolbachia?* One potential risk is that the memory loss induced by *Wolbachia* infection may lead the wasp fail to locate host eggs or habitat plants, as many parasitoid species can learn to associate novel cues with the presence of hosts or nectariferous plants (22). In addition, the intraspecific competition is predicted to reduce the fitness of surviving wasps, such as a lower fecundity and shorter longevity (28). These fitness costs may influence vertical transmission by affecting the reproductive ability of offspring wasps. The costs or benefits of superparasitism for *Wolbachia* should be quantified properly to explain the epidemic of *Wolbachia* and Trichogramma populations. A second question is that as *Wolbachia* infection causes memory loss in Trichogramma wasps, and reduces the ability of host discrimination, do these infected wasps exhibit a high tendency to accept hosts that have been parasitized by different parasitoid species, and gain the opportunity of HT among sympatric parasitoid species? The HT of parthenogeneis-inducing *Wolbachia* between different Trichogramma species probably occurs in nature, though the interspecific HT is rarely reported in the field (9). A previous study reported the natrual interspecific HT of PI-*Wolbachia* between T. deion and T. kaykai (9). T. dendrolimi wasps can parasitize the eggs of many lepidopteran species (30), and may compete for host resources with different egg parasitoid species in different seasons. However, it is still unclear how common such interspecific HT is in nature.

In conclusion, our results enrich the evidence indicating the behavioral mechanisms for host manipulation in *Wolbachia* and support the “host manipulation hypothesis” based on Poulin’s criterion 1–4 and on the observations of previous studies (10, 19, 20, 27). However, as the tests were conducted under laboratory conditions, further studies should be encouraged to test the effect of the behavioral manipulation of *Wolbachia* on the epidemic of *Wolbachia* and the dynamic coexistence of both infected and uninfected populations of Trichogramma in fields.

## MATERIALS AND METHODS

### Insects.

All insects, including a *Wolbachia*-uninfected bisexual isofemale line (TD) and a *Wolbachia*-infected thelytokous isofemale line (TDW) of T. dendrolimi, and their host C. cephalonica were reared under the following conditions: 26 ± 1°C temperature, 75% ± 5% RH, and a 16 h/8 h light/dark cycle. The TDW line, which has the same genetic background as the TD line, was established by artificially transferring *Wolbachia* into females of the TD line (7). *Wolbachia* infection in the TDW line was detected by amplifying the *Wolbachia* surface protein gene (*wsp*) according to the protocol described in our previous studies (10, 20). The *Wolbachia*-uninfected population of C. cephalonica was reared on a semi-artificial diet. The C. cephalonica eggs were glued onto a card using gum arabic. These egg cards were used for parasitization by T. dendrolimi.

### Conditioning procedure.

The conditioning procedures of TD and TDW females were conducted according to the methods described by Farahani et al. (27) and Smid et al. (35). The TD or TDW naive females (emerged within 24 h), in a group of 100 individuals, were supplied with an egg card with ca. 200 host eggs for 30 min to obtain oviposition experience. These wasps were then individually transferred into a training pipe (Diameter = 25 mm, Length = 95 mm) with a conditioning odor at an airflow of 1 m/s and an egg card with 200 host eggs as a reward, and lasted for 120 min. Lemon odor and peppermint odor were used as the conditioning odor. The tested odor was presented on a circular filter paper (Diamter = 20 mm) with 1 μL peppermint or lemon solution (99% pure) in a pipe (Diameter = 20 mm, Length = 70 mm) connected with the training pipe.

Half of the wasps in each group underwent the training procedure using lemon odor, while the other half was trained using peppermint odor. The procedure described above was defined as a single conditioning event. To obtain experienced females with a long-term memory, TD or TDW females were subjected to 3 conditioning events with an interval of 30 min between events.

### Bioassay method.

To test the memory capacity of Trichogramma females after condition, a four-quadrant olfactometer was used to test the preference of Trichogramma females to the conditioning odor. The experiments were conducted in a climate room (26 ± 1°C) with scattered light (2000 lx). The device contained an empty square room space (100 mm × 100 mm) divided into 4 equally shaped areas. Each area was connected to a Teflon capillary holder containing activated carbon and the capillaries to supply conditioning or control odor. The conditioning odor (1 m/s) was pumped into the area from two tunnels placed opposite to each other along a diagonal line. The control odor (clean air) was pumped from the other 2 tunnels. To reduce the effects of previous trials, the device was cleaned with distilled water after each trial. After each trial, the conditioning or control odor in a tunnel was altered by counterpart odor, and the device was rotated clockwise at 90°.

In a trial, a single female was introduced from the hole at the center of the area. The female wasp was then allowed to walk freely in the area. The trial was stopped when the wasps made a choice, or the time reached 900 s. The choice of the wasp was determined when the wasp crossed a line located 30 mm away from a tunnel pumping conditioning or control odor. The residence time of the wasp was defined as the amount of time spent in the area with conditioning or control odor before the wasp crossed the line. The wasps that held still for 180 s were abandoned, and excluded from analysis. The wasps used for different treatment levels were obtained from the same generation of the Trichogramma populations.

### Effects of *Wolbachia* infection and memory blocking on memory of Trichogramma females.

To reduce side effects of ANI and ACD on the survival of Trichogramma females, a 15% honey solution mixed with 0.008 mg/mL ACD or 0.1 mg/mL ANI was selected to block the memory of Trichogramma females according to the results of the toxicology test (Text S1 and Fig. S1). The effective memory durations of experienced female wasp in TD or TDW line was determined by the preference to the odor at 12 h, 24 h, and 36 h after conditioning according to the procedure of bioassay in a four-quadrant olfactometer described above. Compared with experienced females in the TDW line, experienced females in the TD line exhibited a higher tendency to the conditioning odor at 24 h after conditioning. Naïve females did not display a significant difference in the preference between the conditioning odor and control air (Text S1, and Fig. S2 and S3).

10.1128/mbio.02362-22.1 TEXT S1 Effective memory duration of experienced females to the conditioning odor, and side effects of ACD or ANI on the longevity of female wasps in TD and TDW lines. Download Text S1, DOCX file, 0.02 MB. Copyright © 2022 Zhou et al. 2022 Zhou et al. https://creativecommons.org/licenses/by/4.0/ This content is distributed under the terms of the Creative Commons Attribution 4.0 International license.

In this experiment, Trichogramma females subjected to 4 different treatments were used: (i) Naive females without training; (ii) Experienced females that were conditioned with lemon odor or peppermint odor; (iii) Experienced females fed a honey solution mixed with ACD; (iv) Experienced females fed honey solution mixed with ANI. After a 24 h time interval, 60 naive females, experienced females, and experienced females fed ACD or ANI of each Trichogramma line (i.e., 30 females for lemon odor and 30 females for peppermint) were tested according to the procedure of bioassay in a four-quadrant olfactometer described above. The experiments were conducted from 7 am to 11 am on a day, and were accomplished within 5 days. The entire experiments were replicated using different individuals.

### Effects of *Wolbachia* infection and memory blocking on expression of *CREB1* and *PKA*.

The orthologs of *CREB1* and *PKA* were identified by aligning the sequences of T. dendrolimi genomic assembly (Text S2, Table S1 and Fig. S4 and S5). The available CREB1 protein sequence from D. melanogaster (Q9VWW0), N. vitripennis (XP_032455913), Apis mellifera (CAD23075) and Bombyx mori (ADM32514), along with the available PKA protein sequence from D. melanogaster (CAA34841), N. vitripennis (NP_001164381), A. mellifera (XP_026295426) and B. mori (NP_001104823) were used as queries. The primers for *CREB1* and *PKA* were designed using the Primer Premier 5.0 software (Premier Biosoft) (Table S2).

10.1128/mbio.02362-22.2 TEXT S2 Sequences of *CREB1* and *PKA* gene. Download Text S2, DOCX file, 0.01 MB. Copyright © 2022 Zhou et al. 2022 Zhou et al. https://creativecommons.org/licenses/by/4.0/ This content is distributed under the terms of the Creative Commons Attribution 4.0 International license.

10.1128/mbio.02362-22.8 TABLE S1 GenBank accession of *CREB1* and *PKA* used for aligning of sequences. Download Table S1, DOCX file, 0.01 MB. Copyright © 2022 Zhou et al. 2022 Zhou et al. https://creativecommons.org/licenses/by/4.0/ This content is distributed under the terms of the Creative Commons Attribution 4.0 International license.

10.1128/mbio.02362-22.9 TABLE S2 The primers of *CREB1*, *PKA*, and *RPL-18*. Download Table S2, DOCX file, 0.01 MB. Copyright © 2022 Zhou et al. 2022 Zhou et al. https://creativecommons.org/licenses/by/4.0/ This content is distributed under the terms of the Creative Commons Attribution 4.0 International license.

10.1128/mbio.02362-22.6 FIG S4 Amino acid alignments of *CREB1* of different insect species. Grey area indicates the pKID domains and bZIP domains. Download FIG S4, TIF file, 1.1 MB. Copyright © 2022 Zhou et al. 2022 Zhou et al. https://creativecommons.org/licenses/by/4.0/ This content is distributed under the terms of the Creative Commons Attribution 4.0 International license.

10.1128/mbio.02362-22.7 FIG S5 Amino acid alignments of *PKA* of different insect species. Grey area indicates the SKTc_PKA domains. Download FIG S5, TIF file, 1.2 MB. Copyright © 2022 Zhou et al. 2022 Zhou et al. https://creativecommons.org/licenses/by/4.0/ This content is distributed under the terms of the Creative Commons Attribution 4.0 International license.

The TD or TDW 1-day-old females fed honey solution only, ACD, or ANI, in groups of 100 offspring individuals, were collected and stored at −80°C. Each treatment involved 24 replicates. The entire experiments were replicated using different individuals. The total RNA content of these females was extracted using TRIzol reagent (Ambion, Life Technologies) according to the protocol described in our previous study (52). The RT-qPCRs were carried out in the Bio-Rad CFX96 Real-time PCR Detection System (Bio-Rad, Hercules,) according to the procedure described in our previous study (52). The expressions of *CREB1* and *PKA* were normalized using a reference gene, *RPL18*, which is considered an appropriate reference gene for the adult stage of T. dendrolimi (52).

### Effects of *Wolbachia* infection and memory blocking on superparasitism of Trichogramma females.

To obtain the oviposition experience, TD or TDW naive females (emerged within 24 h) were individually supplied with a card with 10 host eggs for 1 h. Thirty of each of naive females, experienced females fed honey solution only, experienced females fed ACD, and experienced females fed ANI, of TD and TDW lines, were supplied with an egg card with 10 host eggs for 1 h after 24 h. The oviposition behaviors were recorded under the stereo zoom microscope (Zeiss SV6). Though a T. dendrolimi female often deposit one offspring egg into a C. cephalonica egg by a time of oviposition, a few host eggs may not have contained an offspring egg or may have contained 2 offspring eggs after a single oviposition. A single egg from a female wasp was deposited into a host egg only when a single oviposition behavior occurred with fluctuating abdominal movements (29, 53, 54). The location of the parasitized host egg in the egg card was marked by a surgical skin marking pen (0.5 mm tips, T3023; Tondaus) soon after a trial. The host eggs were maintained until the eggs blackened, indicating the parasitization of the host egg (55). The blackened eggs were cut off from the egg card and individually transferred into a new Durham glass tube for emergence. After the emergence of the wasp offsprings, the host eggs were dissected to determine the presence of dead Trichogramma offsprings. The superparasitized host eggs were defined as eggs with 2 Trichogramma offspring deposited by two times of oviposition (28). The entire experiments were replicated using different individuals.

### FISH detection of *Wolbachia* in eggs, larvae, prepupae, pupae, and adults of Trichogramma.

To investigate the presence of *Wolbachia* in the neurotrophic part of Trichogramma, the fluorescence *in situ* hybridization (FISH) was applied to detect *Wolbachia* signals in the eggs, larvae, pre-pupae, pupae, and adult wasps of Trichogramma. The procedure of FISH detection was conducted according to the description of Zhao et al. (56) and Wang et al. (57). A group of ca. 500 TD or TDW females were transferred into a glass tube (75 mm length × 12 mm diameter) containing the egg card with ca. 200 host eggs for 10 min. The eggs were individually cut out from the cards and then dissected at 0.5 h (egg stage), 48 h (larval stage), 96 h (prepupal stage), 144 h (pupal stage), 240 h (newly emerged adult) after parasitization under a stereomicroscope (SV6; Zeiss). To target the 16S rRNA (rRNA) of *Wolbachia*, two 5′ rhodamine-labeled *Wolbachia* probes were used (W1, 5′- AATCCGGCCGAACCGACCC-3′ and W2, 5′- CTTCTGTGAGTACCGTCATTATC-3′) (58). The location of *Wolbachia* in the *Trichogramma* offspring or adults was photographed under a laser scanning confocal microscope (Olympus FV3000, Monolith). The images in Fig. 5 are representative images of 16 eggs, 10 larvae, 10 pre-pupae, 10 pupae, and 10 adults. The entire experiments were replicated using different female individuals.

### Data analysis.

To examine memory inhibition in TD or TDW females fed ACD or ANI, the GLM with quasi-binomial distribution was used to analyze the PCO and PRO, with 24 h time interval after conditioning in naive females and experienced females fed honey solution only, ACD, or ANI. Exact binomial tests were applied to test PCO and PRO against the theoretical value of 50%. To test the effects of memory inhibition and *Wolbachia* infection on the superparasitism tendency of *Trichogramma* females, the frequency of superparasitism by TD or TDW females fed honey solution only, ACD, or ANI was also analyzed using GLM with quasi-binomial distribution as well. The GLM with Gaussian distribution was applied to analyze the expression of memory-related genes, *CREB1* and *PKA*, in TD or TDW females fed honey solution only, ACD, or ANI. All data calculations and analyses were conducted using R software version. 4.0.2 (57).
