# Supplementary figures and images for: Wolbachia-Driven Memory Loss in a Parasitic Wasp Increases Superparasitism to Enhance Horizontal Transmission
Source: mBio. 2022 Oct 10;13(6):e02362-22. doi: 10.1128/mbio.02362-22 (PMC9765423; doi:10.1128/mbio.02362-22)

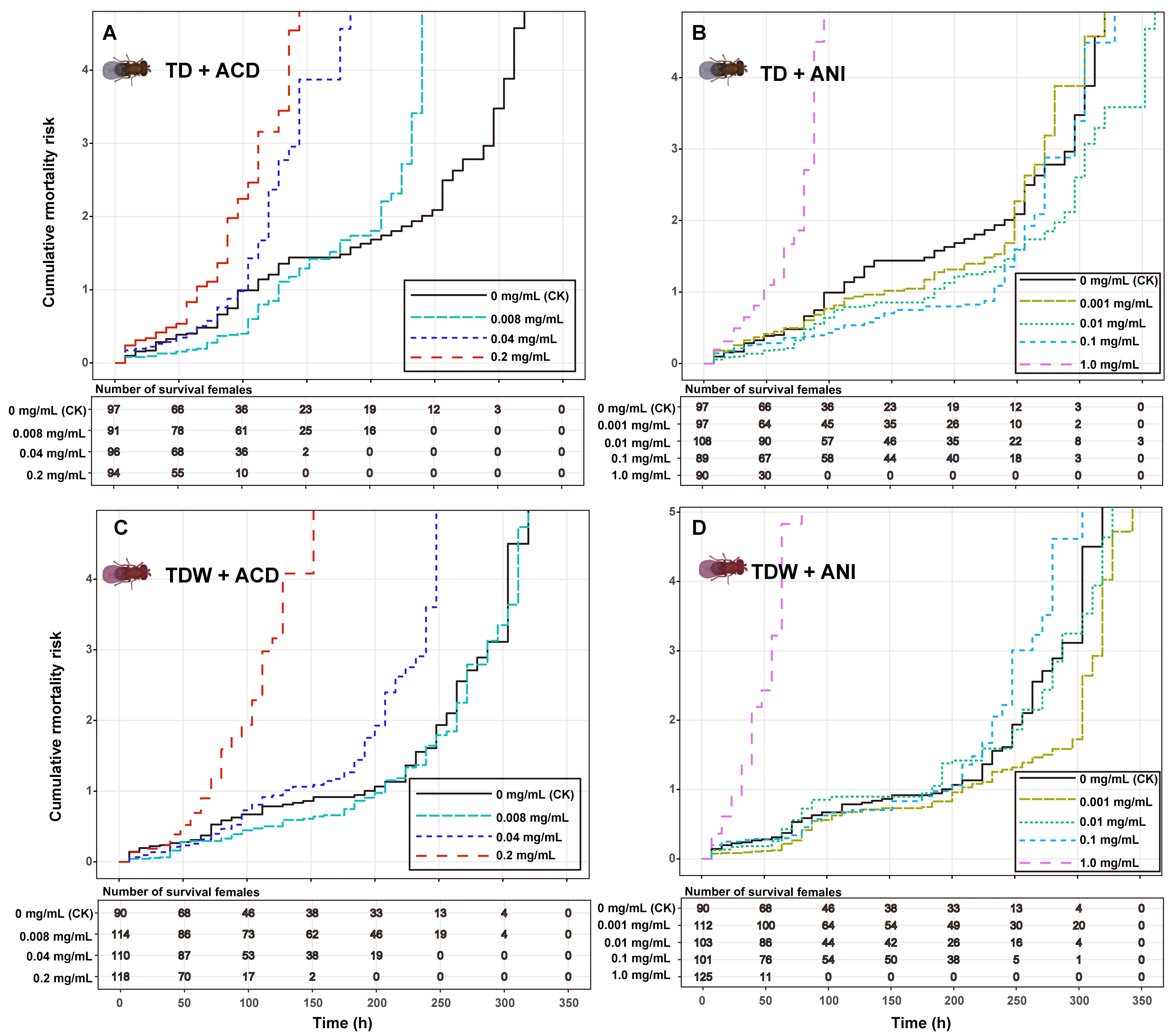

Supplement: FIG S1 [file mbio.02362-22-s0003.tif]

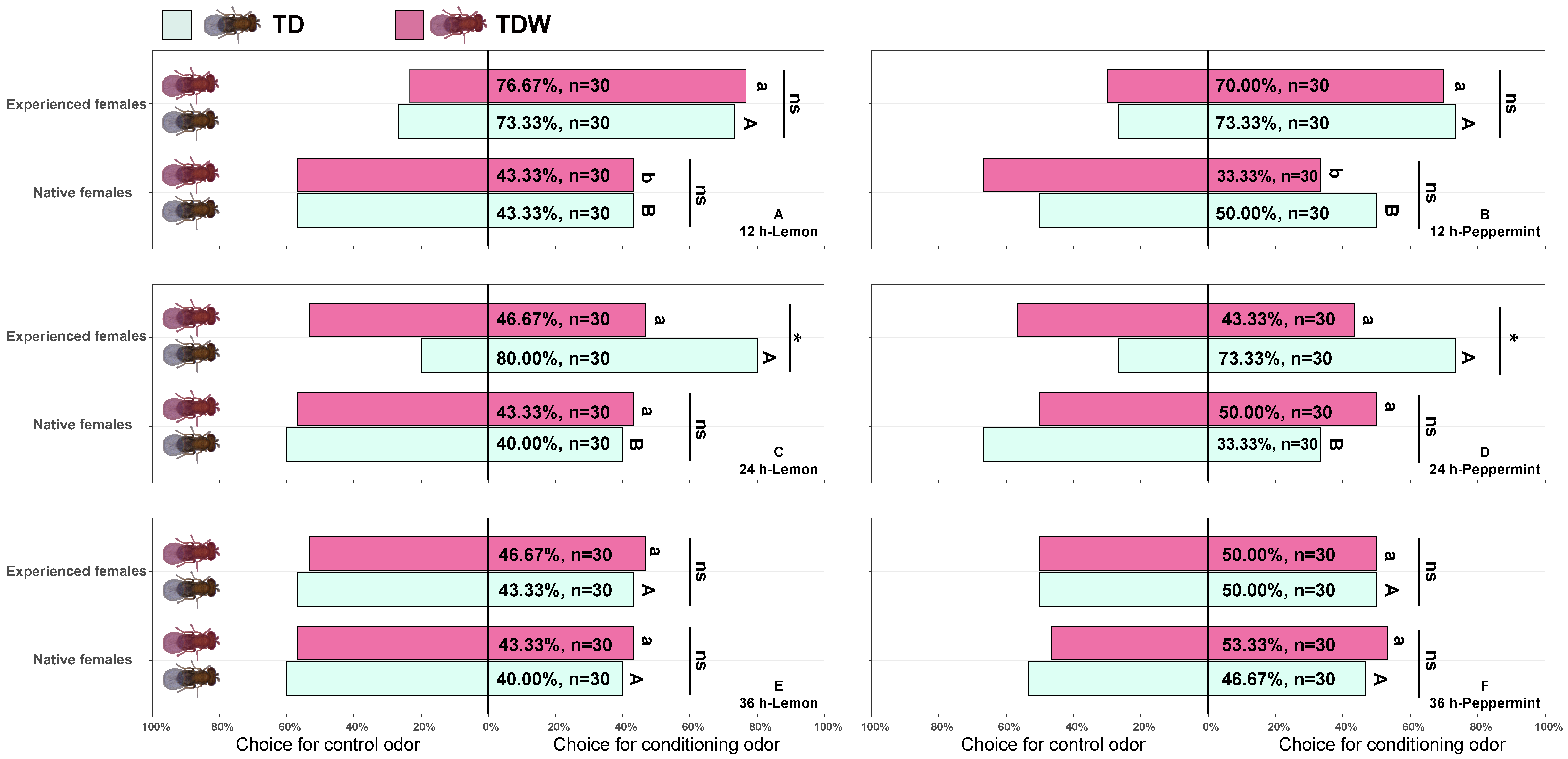

Supplement: FIG S2 [file mbio.02362-22-s0004.tif]

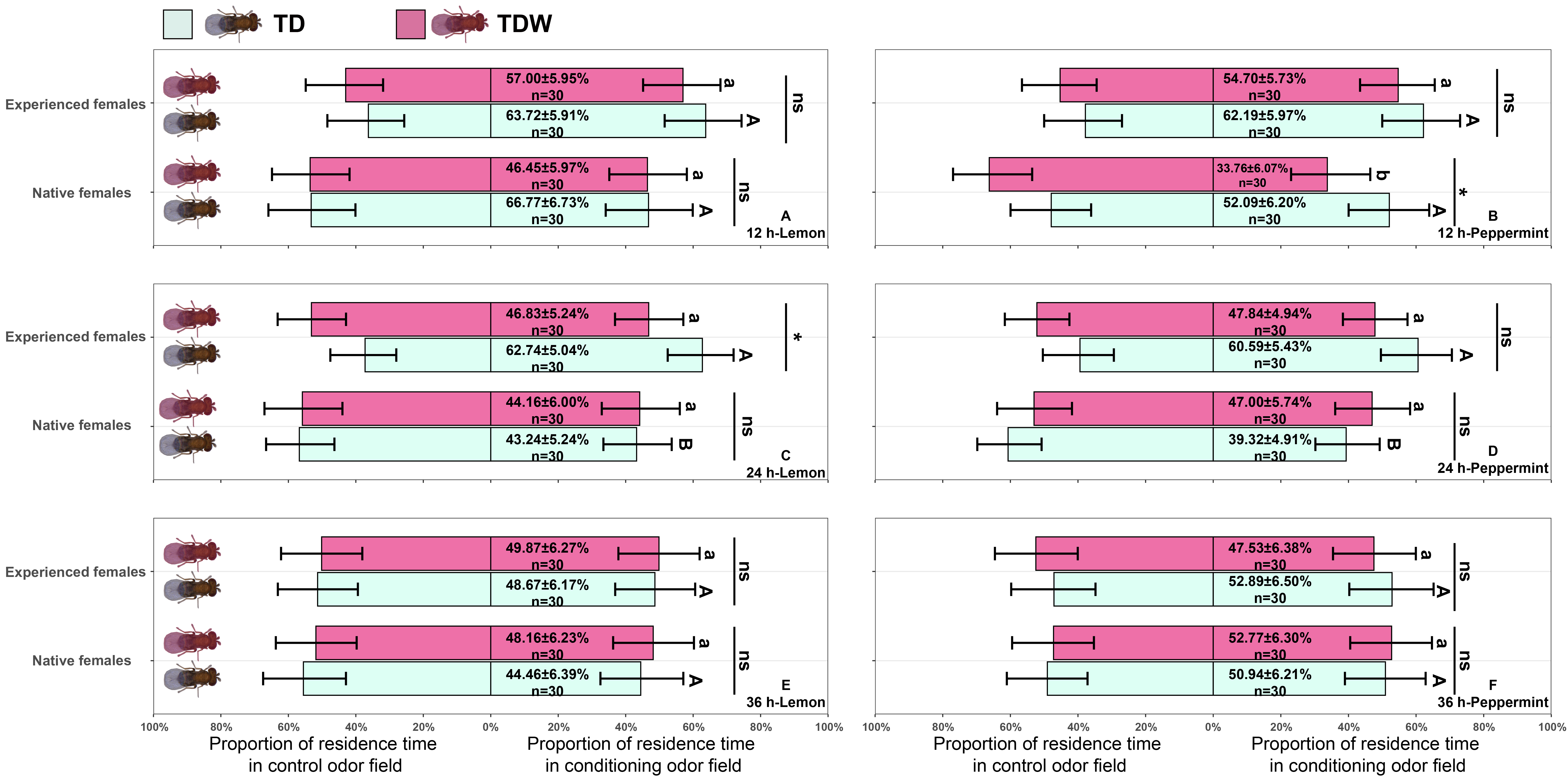

Supplement: FIG S3 [file mbio.02362-22-s0005.tif]

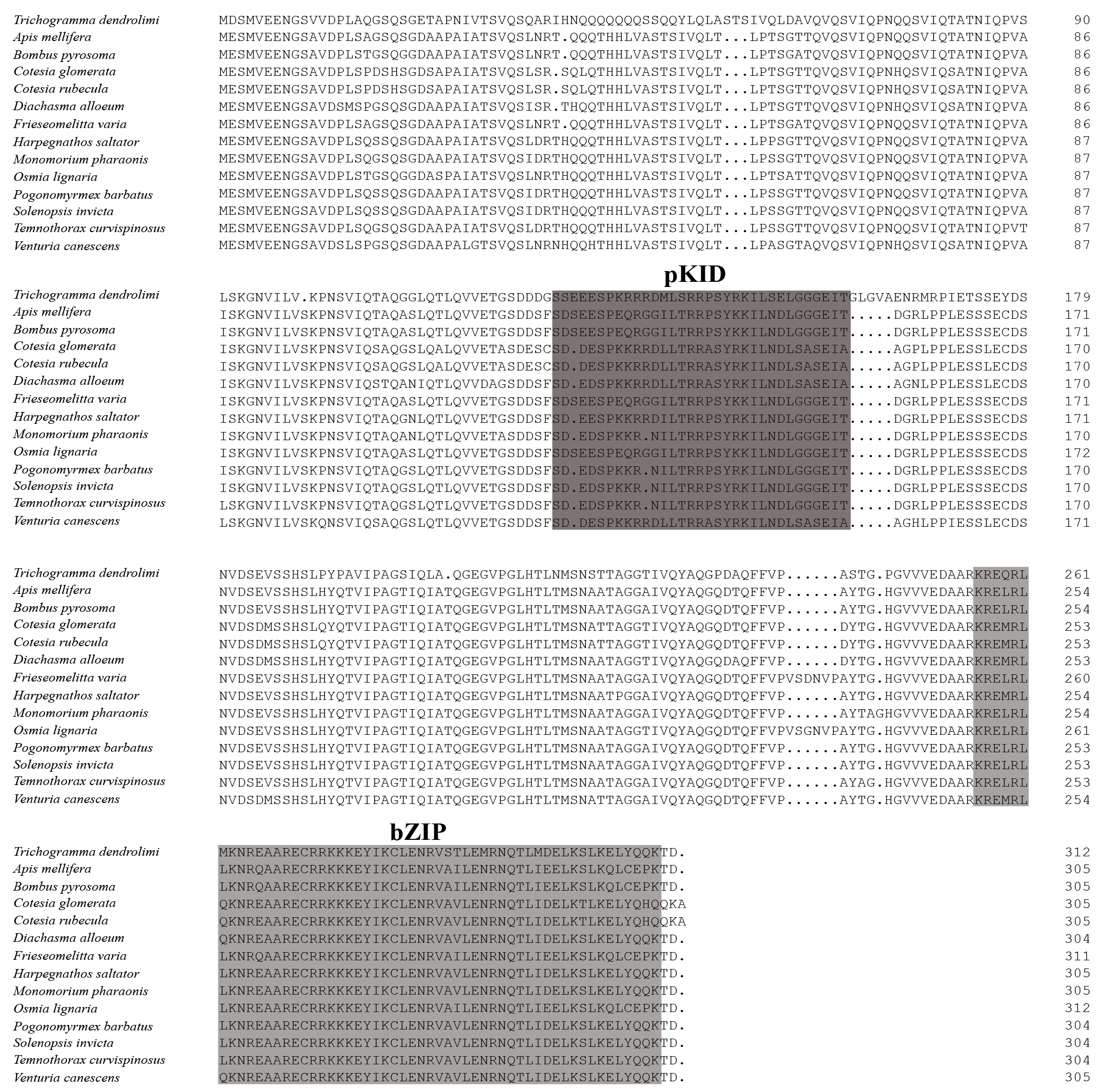

Supplement: FIG S4 [file mbio.02362-22-s0006.tif]

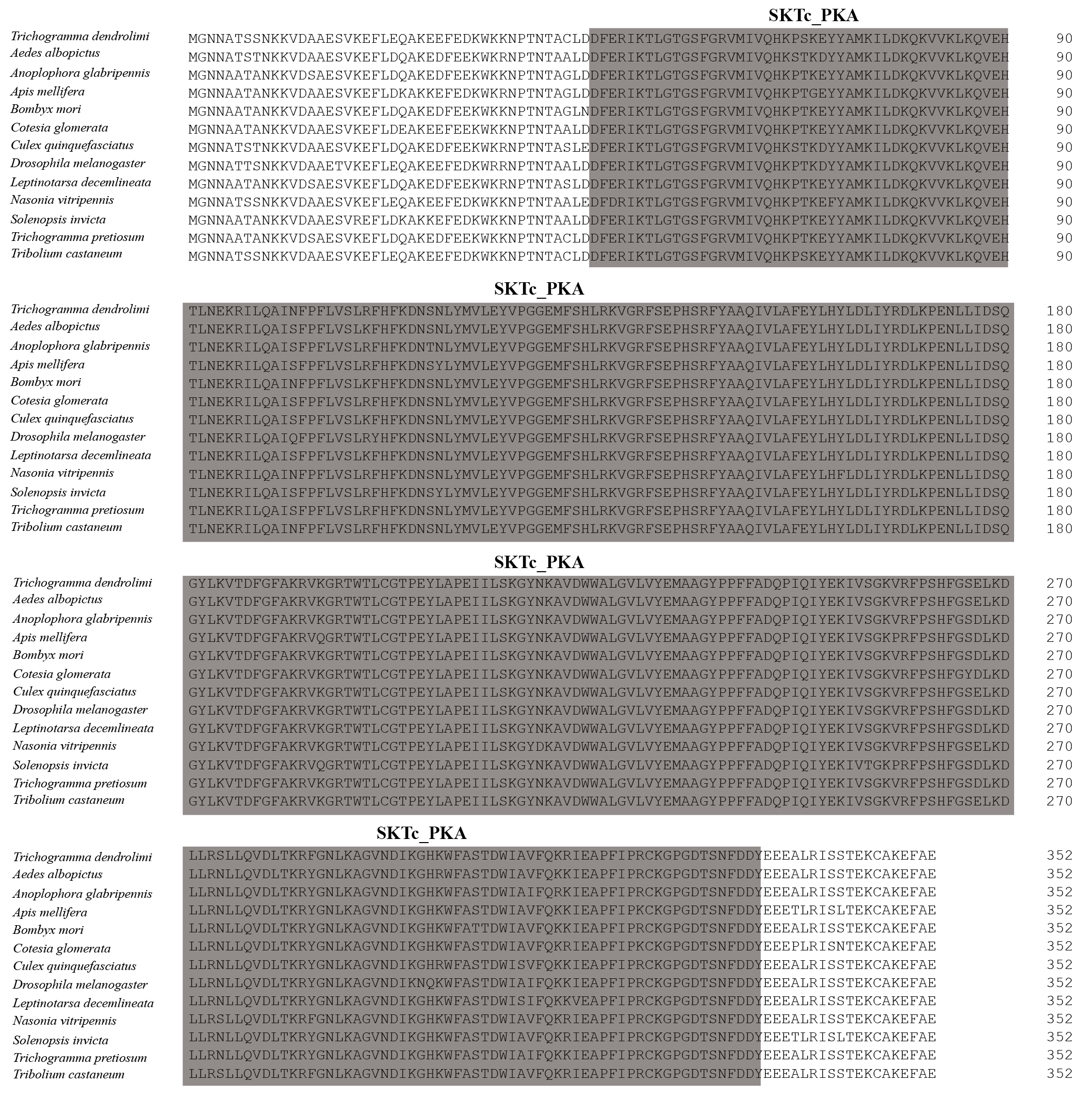

Supplement: FIG S5 [file mbio.02362-22-s0007.tif]
