# Supplementary material for: Wolbachia-Driven Memory Loss in a Parasitic Wasp Increases Superparasitism to Enhance Horizontal Transmission
Source: mBio. 2022 Oct 10;13(6):e02362-22. doi: 10.1128/mbio.02362-22 (PMC9765423; doi:10.1128/mbio.02362-22)
Supplement: TEXT S1 [file mbio.02362-22-s0001.docx]

**Effective memory duration of experienced females to the conditioning odor and side effects of ACD or ANI on the longevity of female wasps in TD and TDW lines**

1. **Materials and Methods**

To block long-term memory formation without affecting the learning and survival of *Trichogramma* females, the females were fed a 15% honey solution mixed with different concentrations of the memory inhibitor, ACD or ANI. To reduce side effects of ANI and ACD on the survival of *Trichogramma* females, TD or TDW females were fed a 15% honey solution mixed with 0.008, 0.04, or 0.2 mg/mL ACD, or 0.001, 0.01, 0.1, or 1.0 mg/mL ANI for 2 h. The TD or TDW females fed honey solution only were used as the control groups. Thereafter, to analyze the effects of ACD or ANI on the longevity of the parasitoids, the treated *Trichogramma* females were individually transferred into a Durham glass tube and fed daily with 15% honey solution till death. The longevity of each female was checked every 8 h and recorded.

To test the effective memory duration of native and experienced females in TD and TDW lines, both groups were individually transferred into an empty glass tube for 12 h, 24 h, and 36 h. The time intervals after conditioning were set based on the results of Farahani et al. (1). For each time interval, 60 experienced or native females of each *Trichogramma* line (i.e., 30 females for lemon odor and 30 females for peppermint) were tested according to the procedure of bioassay in a four-quadrant olfactometer described above. The entire experiments were replicated using different individuals.

1. **Data analysis**

To select the dosage of ACD and ANI that has fewer side effects on the survival of TD or TDW females, the Cox’s proportional hazard model (Cox model) was applied to compare the longevity of females fed honey solution only (the control group, CK) and that of females fed different doses of ACD or ANI. The Cox model quantified the longevity based on the hazard rate, which indicated the death risk of wasps at different ages. The hazard death rate *h(t)* at age *t* is given by:

$$h(t)=h_{0}(t)\exp\left\{\sum_{i=1}^{n} \beta_{i}z_{i}\right\}$$

where *h_0_(t)* is the baseline hazard function when *z_i_* is zero, and *βi* is the regression coefficient. If the hazard ratio, expressed by exp｛Σ*β_i_Z_i_*｝, is decreased, the longevity will be increased (2, 3).

To examine the effective memory duration of experienced and native females of TD or TDW line, the generalized linear model (GLM) with binomial distribution was applied to analyze the proportion of choice (PCO) and residence time (PRO) for the conditioning odor, with 12, 24, and 36 h time interval after learning. Exact binomial test was applied to test PCO and PRO against the theoretical value of 50%.

All data calculations and analyses were conducted by using R software ver. 4.0.2 (4).

1. **Results**

**3.1 Effects of ACD and ANI on the longevity of TD or TDW females**

The longevity of females fed honey solution only was significantly higher than that of those fed 0.2 mg/mL (TD: Coefficient ± SE = 1.20 ± 0.16, *β* = 3.34, *z* = 7.36, *P* < 0.001; TDW: 1.41 ± 0.16, *β* = 4.11, *z* = 8.82, *p* < 0.001) and 0.04 mg/mL of ACD (TD: 0.71 ± 0.16, *β* = 2.03, *z* = 4.43, *p* < 0.001; TDW: 0.50 ± 0.15, *β* = 1.64, *z* = 3.33, *p* = 0.0048), but was not higher than that of those fed 0.008 mg/mL of ACD (TD: 0.022 ± 0.15, *β* = 1.023, *z* = 0.15, *p* = 0.999; TDW: -0.13 ± 0.14, *β* = 0.87, *z* = 0.95, *p* = 0.78)(Fig. S1 A, C).

The longevity of females fed honey solution only was significantly higher than that of those fed 1.0 mg/mL of ANI (TD: 1.27 ± 0.16, *β* = 3.56, *z* = 7.97, *p* < 0.001; TDW: 2.15 ± 0.17, *β* = 8.56, *z* = 12.47, *p* < 0.001), but was not higher than that of those fed 0.1 (TD: -0.36 ± 0.15, *β* = 0.70, *z* = 2.41, *p* = 0.11; TDW: 0.12 ± 0.15, *β* = 1.12, *z* = 0.80, *p* = 0.93), 0.01 (TD: -0.42 ± 0.14, *β* = 0.66, *z* = 2.08, *p* = 0.23; TDW: 0.025 ± 0.14, *β* = 1.03, *z* = 0.17, *p* = 0.9998), and 0.001 mg/mL of ANI (TD: -0.051 ± 0.14, *β* = 0.95, *z* = 0.35, *p =* 0.997; TDW: -0.40 ± 0.14, *β* = 0.67, *z* = 2.78, *p* = 0.043)(Fig. S1 B, D).

**3.2 Memory duration of native and experienced females**

After conditioning and a 12 h time interval, the PCOs of experienced TD females (*p* = 0.0052 [Lemon]; *p* = 0.043 [Peppermint]) and TDW females (*p* = 0.016 [Lemon]; *p* = 0.016 [Peppermint]) were significantly higher than the theoretical value of 50%, but these were not true in case of naïve TD females (*p* = 0.58 [Lemon]; *p* = 0.58 [Peppermint]) and naïve TDW females (*p* = 0.099 [Lemon]; *p* = 1.00 [Peppermint]). After conditioning and a 24 h time interval, the PCOs of experienced TD females were significantly higher (*p* = 0.0014 [Lemon]; *p* = 0.016 [Peppermint]) than the theoretical value of 50%, but these were not true in case of experienced TDW females (*p* = 0.86 [Lemon]; *p* = 0.58 [Peppermint]). After conditioning and a 36 h time interval, the PCOs of naïve females and experienced females in TD and TDW lines were not different to theoretical value of 50%. Regardless of *Trichogramma* line and time interval, the PROs of naïve females and experienced females were not different to theoretical value of 50%.

After conditioning and a 12 h time interval, the PCOs of experienced females were significantly higher (TD females: *z* = 2.27, *p* = 0.023 [Lemon]; *z* = 2.15, *p* = 0.032 [Peppermint]; TDW females: *z* = 2.53, *p* = 0.012 [Lemon]; *z* = 2.73, *p* = 0.0064 [Peppermint]) than those of native females, regardless of the *Trichogramma* line and conditioning odor. In experienced or native females conditioned with lemon or peppermint odor, the differences in PCO between TD and TDW females were insignificant (Native females: *z* = 0, *p* = 1.00 [Lemon]; *z*= 1.28, *p* = 0.20 [Peppermint]; Experienced females: *z* = 0.29, *p* = 0.77 [Lemon]; *z* = 0.28, *p* = 0.78 [Peppermint])(Fig. S2 a, b). Regardless of the *Trichogramma* line and conditioning odor, the PROs of experienced females were insignificantly (TD females: *z* = 1.86, *p* = 0.063 [Lemon]; *z* = 1.17, *p* = 0.24 [Peppermint]; TDW females: *z* = 1.24, *p* = 0.51 [Lemon]) or significantly (TDW females: *z* = 2.42, *p* = 0.016 [Peppermint]) higher than those of native females. Except for the PRO of native females with peppermint odor, the differences in PRO between TD and TDW females were insignificant (Native females: *z* = 0.035, *p* = 0.97 [Lemon]; *z* = 2.06, *p* = 0.040 [Peppermint]; Experienced females: *z* = 0.80, *p* = 0.42 [Lemon]; *z* = 0.90, *p* = 0.37 [Peppermint])(Fig.S3 A, B).

After conditioning and a 24 h time interval, the PCOs of experienced TD females were significantly higher than those of native TD females (*z* = 2.99, *p* = 0.0028 [Lemon]; *z* = 2.96, *p* = 0.031 [Peppermint]), but these differences were not true for TDW females (*z* = 0.26, *p* = 0.80 [Lemon]; *z* = 2.73, *p* = 0.0064 [Peppermint])(Fig. S2 d, e). The PROs of experienced TD females were significantly higher than those of native TD females (*z* = 2.61, *p* = 0.0090 [Lemon]; *z* = 2.82, *p* = 0.0049 [Peppermint]), but these differences were not true for TDW females (*z* = 0.33, *p* = 0.74 [Lemon]; *z* = 0.11, *p* = 0.91 [Peppermint])(Fig. S3 D, E).

After conditioning and a 36 h time interval, the PCOs and PROs of females were not affected by the conditioning, the *Trichogramma* line, and the interaction of the conditioning and *Trichogramma* line (Fig. S2 E, F ; Fig. S3 E, F).

**REFERENCES**

1. Farahani HK, Ashouri A, Goldansaz SH, Shapiro MS, Pierre JS, Van Baaren J. 2017. Decrease of memory retention in a parasitic wasp: an effect of host manipulation by *Wolbachia*? Insect Sci 24: 569-583. <https://doi.org/10.1111/1744-7917.12348>.
2. Cox DR. 1972. Regression models and life-tables. J Roy Stat Soc 74: 187-120. <https://doi.org/10.1111/j.2517-6161.1972.tb00899.x.>
3. Zhou JC, Zhao Q, Liu SM, Shang D, Zhao X, Huo LX, Dong H, Zhang LS. 2021. Effects of thelytokous parthenogenesis-inducing *Wolbachia* on the fitness of *Trichogramma dendrolimi* Matsumura (Hymenoptera: Trichogrammatidae) in superparasitised and single-parasitised hosts. Front Ecol Evol 9:730664. <https://doi.org/10.3389/fevo.2021.730664>
4. R Development Core Team. 2020. R: A language and environment for statistical computing. R Foundation for Statistical Computing, Vienna, Austria. URL <https://www.R-project.org/.>
