# Supplementary material for: Wolbachia-Driven Memory Loss in a Parasitic Wasp Increases Superparasitism to Enhance Horizontal Transmission
Source: mBio. 2022 Oct 10;13(6):e02362-22. doi: 10.1128/mbio.02362-22 (PMC9765423; doi:10.1128/mbio.02362-22)
Supplement: TEXT S2 [file mbio.02362-22-s0002.docx]

CREB1

ATGGACAGCATGGTCGAGGAGAACGGATCAGTTGTGGACCCACTTGCGCAGGGATCGCAG

AGCGGCGAGACCGCCCCCAACATCGTGACATCGGTACAGTCTCAAGCTAGGATACACAAT

CAGCAACAACAACAACAACAATCATCCCAACAATATCTACAGCTTGCCTCTACCAGCATT

GTGCAACTCGACGCAGTGCAGGTCCAATCAGTTATTCAACCAAATCAACAGTCAGTTATT

CAAACTGCCACAAATATACAACCTGTATCTCTTTCTAAAGGAAATGTTATTCTTGTCAAA

CCCAACTCTGTTATTCAGACAGCTCAAGGAGGCTTACAGACATTACAAGTTGTCGAAACT

GGCAGTGATGATGATGGTAGCTCTGAAGAAGAATCTCCAAAAAGAAGAAGAGATATGCTA

TCGCGTAGGCCATCATATAGGAAAATACTAAGTGAATTAGGCGGTGGAGAAATAACTGGA

TTAGGTGTTGCAGAGAATCGTATGCGACCAATTGAAACTTCTTCTGAATATGATTCTAAT

GTGGATAGCGAAGTATCTTCTCACTCGTTACCCTATCCTGCAGTTATACCAGCAGGATCA

ATACAATTAGCTCAAGGAGAAGGAGTACCAGGACTACACACCTTAAATATGAGTAATTCA

ACAACAGCAGGTGGCACAATAGTACAATATGCTCAAGGTCCAGATGCACAATTCTTCGTA

CCAGCTAGTACTGGCCCTGGAGTAGTTGTCGAAGATGCAGCAAGAAAACGAGAACAGAGG

CTGATGAAAAATAGAGAGGCTGCTCGAGAGTGTAGAAGAAAAAAGAAAGAATATATAAAG

TGTTTAGAAAATCGTGTGAGTACACTTGAAATGAGGAATCAAACTTTAATGGATGAACTT

AAATCGTTAAAAGAATTATACCAGCAGAAAACGGATTGA

PKA

ATGGCTGCGACGACGACTGCTGCTCTCGCTACTGCTAAAAAGGCGACGACAACCAACGCG

ACGACGCTAATGATGATGACTTCGATGATCGAGTTCGATCTCGCTAGCAGTACGATGAGC

ACCGACTGTTTTACGGCGCGATTAACGAAAAAAGCCAGCGCAATTCTGAGAAAACTCGAC

ATCCGTGGAGCCCTGGCTGGCTCGAGCGGCTCCAACTCGTCGTCGTCAGCAGCAGCAGCA

GCAGCAGGTAGCAGCGGGGCCGCCTCCACCTCGGGCAGCGGCAAAATGGGCAACAATGCA

ACGAGCTCCAACAAAAAGGTCGACGCGGCCGAGAGCGTCAAGGAGTTCCTCGAGCAGGCC

AAGGAGGAGTTCGAGGACAAATGGAAGAAGAACCCGACCAACACGGCGTGCCTCGACGAC

TTCGAGCGCATCAAGACCCTCGGCACCGGCTCCTTCGGACGCGTCATGATAGTCCAGCAC

AAGCCGTCCAAGGAGTACTACGCCATGAAGATACTCGACAAGCAGAAAGTCGTCAAGCTC

AAGCAGGTCGAGCACACACTCAACGAAAAACGCATTCTTCAAGCCATCAATTTTCCTTTC

CTCGTCTCGCTTCGTTTTCACTTTAAGGATAATTCTAATCTCTACATGGTACTCGAGTAC

GTTCCTGGAGGTGAAATGTTCAGCCATCTCAGGAAAGTTGGCCGCTTCTCTGAGCCACAC

TCCAGGTTCTATGCTGCTCAGATTGTGCTGGCCTTTGAGTATCTCCATTACTTAGATCTT

ATTTATAGAGATCTCAAGCCTGAAAATCTGCTGATAGATTCGCAAGGTTATCTCAAGGTC

ACAGATTTTGGCTTCGCTAAGAGAGTCAAGGGTAGAACGTGGACCCTATGCGGTACACCA

GAGTATCTAGCCCCTGAAATTATTCTCAGCAAAGGTTACAACAAAGCTGTGGATTGGTGG

GCGCTTGGAGTGTTAGTGTATGAAATGGCAGCTGGTTATCCACCGTTCTTCGCTGATCAA

CCTATACAGATTTATGAAAAAATTGTCAGTGGAAAAGTGAGATTTCCATCCCACTTTGGT

TCTGAATTGAAAGACCTTCTACGTAGTTTGCTGCAAGTCGATTTAACCAAGCGATTTGGC

AATCTAAAAGCTGGTGTCAATGATATTAAAGGACACAAGTGGTTTGCCAGCACTGACTGG

ATCGCTGTCTTCCAGAAGCGTATCGAAGCACCCTTTATACCTAGATGTAAGGGTCCAGGA

GATACAAGTAACTTTGATGATTACGAAGAAGAAGCACTGAGAATTTCATCAACTGAGAAA

TGCGCAAAGGAATTTGCTGAATTTTGA
