# Supplementary material for: Wolbachia-Driven Memory Loss in a Parasitic Wasp Increases Superparasitism to Enhance Horizontal Transmission
Source: mBio. 2022 Oct 10;13(6):e02362-22. doi: 10.1128/mbio.02362-22 (PMC9765423; doi:10.1128/mbio.02362-22)
Supplement: TABLE S1 [file mbio.02362-22-s0008.docx]

**Table S1** GenBank accession of *CREB1* and *PKA* used for aligning of sequences

| Gene | Species | GenBank accession no. |
| --- | --- | --- |
| *CREB1* | *Bombyx mori* | ABV48888 |
|  | *Helicoverpa armigera* | ALP86606 |
|  | *Drosophila melanogaster* | AAB35092 |
|  | *Aedes aegypti* | AAL92477 |
|  | *Nasonia vitripennis* | XP_032455913 |
|  | *Lariophagus distinguendus* | ACX54098 |
|  | *Cotesia rubecula* | ACI03623 |
|  | *Cotesia glomerata* | ACI03614 |
|  | *Harpegnathos saltator* | EFN81053 |
|  | *Polyrhachis vicina* | ACB20690 |
|  | *Apis mellifera carnica* | CAD23075 |
|  | *Apis mellifera* | XP_006570114 |
| *PKA* | *Nasonia vitripennis* | NP_001164381 |
|  | *Drosophila melanogaster* | sp P12370.3 |
|  | *Apis mellifera* | XP_393285 |
|  | *Aedes albopictus* | XP_029719295 |
|  | *Tribolium castaneum* | EEZ99369 |
|  | *Cotesia glomerata* | XP_044590468 |
|  | *Culex quinquefasciatus* | EDS26798 |
|  | *Leptinotarsa decemlineata* | XP_023012744 |
|  | *Solenopsis invicta* | XP_039310371 |
|  | *Trichogramma pretiosum* | XP_014226375 |
|  | *Bombyx mori* | NP_001093303 |
|  | *Anoplophora glabripennis* | XP_018565111 |
