# Supplementary material for: Wolbachia-Driven Memory Loss in a Parasitic Wasp Increases Superparasitism to Enhance Horizontal Transmission
Source: mBio. 2022 Oct 10;13(6):e02362-22. doi: 10.1128/mbio.02362-22 (PMC9765423; doi:10.1128/mbio.02362-22)
Supplement: TABLE S2 [file mbio.02362-22-s0009.docx]

Table S2. The primers of *CREB1*, *PKA*, and *RPL-18*

| Gene | Primers |
| --- | --- |
| *CREB1* | Forward: CAACAGCAGGTGGCACAATA |
|  | Reverse: TCATCAGCCTCTGTTCTCGT |
| *PKA* | Forward: ACGCTAATGATGATGACTT |
|  | Reverse: TCATCAGCCTCTGTTCTCGT |
| *RPL-18* | Forward: CGGCCCTTTGATCTGACCAA |
|  | Reverse: TCATCAGCCTCTGTTCTCGT |
